# The impact of *MEIS1* TALE homeodomain transcription factor knockdown on glioma stem cell growth

**DOI:** 10.1080/19768354.2024.2327340

**Published:** 2024-03-13

**Authors:** Hyun-Jin Kim, Don Carlo Batara, Young-Jun Jeon, Seongsoo Lee, Samuel Beck, Sung-Hak Kim

**Affiliations:** a Animal Molecular Biochemistry Laboratory, Department of Animal Science, College of Agriculture and Life Sciences, Chonnam National University, Gwangju, Republic of Korea; b Department of Integrative Biotechnology, Sungkyunkwan University, Suwon-si, Gyeonggi-do, Republic of Korea; c Gwangju Center, Korea Basic Science Institute (KBSI), Gwangju, Republic of Korea; d Department of Systems Biotechnology, Chung-Ang University, Anseong-si, Gyeonggi-do, Republic of Korea; e Department of Dermatology, Center for Aging Research, Chobanian & Avedisian School of Medicine, Boston University, Boston, USA

**Keywords:** *MEIS1*, glioblastoma, glioma stem cell

## Abstract

Myeloid ecotropic virus insertion site 1 (*MEIS1*) is a HOX co-factor necessary for organ development and normal hematopoiesis. Recently, *MEIS1* has been linked to the development and progression of various cancers. However, its role in gliomagenesis particularly on glioma stem cells (GSCs) remains unclear. Here, we demonstrate that *MEIS1* is highly upregulated in GSCs compared to normal, and glioma cells and to its differentiated counterparts. Inhibition of *MEIS1* expression by shRNA significantly reduced GSC growth in both *in vitro* and *in vivo* experiments. On the other hand, integrated transcriptomics analyses of glioma datasets revealed that *MEIS1* expression is correlated to cell cycle-related genes. Clinical data analysis revealed that *MEIS1* expression is elevated in high-grade gliomas, and patients with high *MEIS1* levels have poorer overall survival outcomes. The findings suggest that *MEIS1* is a prognostic biomarker for glioma patients and a possible target for developing novel therapeutic strategies against GBM.

## Introduction

Glioblastoma multiforme (GBM) is classified as a WHO grade IV glioma and is the most malignant type of primary brain tumor. Individuals with GBM have an average overall survival of 15–18 months after diagnosis (Alves et al. 2021; Wu et al. 2021). Despite multimodal treatment options like surgery, radiotherapy, and chemotherapy, current approaches have had extremely limited success (Davis 2016; Taylor et al. 2019; Roh et al. 2023). In recent years, glioma stem cells (GSCs) have been linked to the aggressive characteristics of GBM (Alves et al. 2021). GSCs are identified by their capacity to self-renew and produce more differentiated progenies, which constitute most of the tumor mass (Suvà and Tirosh 2020). GSCs can recapitulate the heterogeneity of the parental tumor, drive resistance to treatments, and survive in harsh and complex microenvironmental niches (Park and Rich 2009; Prager et al. 2020). Maintaining the pluripotency and self-renewal abilities of cancer stem cells (CSC) mostly depends on chromatin regulators, master transcription factors (TFs), and related cellular networks. These regulators frequently act as oncogenes by encouraging the reacquisition of programs necessary for CSC dedifferentiation (Suvà and Tirosh 2020).

The HOX genes encode a family of homeodomain (HD)–containing TFs that play significant functions in embryogenesis and morphogenesis during development and adulthood. These genes are also involved in the formation of segmental identity along the anterior-posterior (AP) body axis, as well as the regulation of cell proliferation, differentiation, and survival (Mallo et al. 2010; Gonçalves et al. 2020; Feng et al. 2021; Arunachalam et al. 2022). Transcriptional regulation, nuclear dynamics, RNA processing, long noncoding RNAs (lncRNAs), miRNAs, and translational processes all influence HOX gene expression (Girgin et al. 2020). Several studies showed that the expression of HOX genes is frequently dysregulated in leukemia, breast, brain, lung, liver, colon, cholangiocarcinoma, pancreatic, skin, head and neck, kidney, bladder, prostate, cervix, endometrial, and ovarian cancer (Brotto et al. 2020; Gonçalves et al. 2020; Feng et al. 2021).

One of the key HD proteins is the triple amino acid loop extension (TALE) homeobox family, which has 27 members (MEIS, PBX, IRX, MKX, PKNOX, TGIF, and pseudogenes) (Girgin et al. 2020). MEIS and related Pre-B-cell Leukemia Homeobox (PBX)-HOX proteins can act as both oncogenes and tumor suppressors in certain biological contexts (Girgin et al. 2020). Co-factors like the PBX form heterodimers with HOX1–9 proteins, while the MEIS family dimerizes with HOX9–13 proteins. The binding of these co-factors improves the attachment and interaction of the highly conserved HD of HOX proteins with the DNA. These co-factors facilitate the recruitment of transcriptional inhibitors such as histone deacetylase (HDAC) and RNA polymerase II or III, leading to differential gene regulation based on the target site's location and sequence in the enhancer or promoter region (Arunachalam et al. 2022).

*MEIS1* plays a crucial role in cellular development and proliferation (Li et al. 2022). The *MEIS1* locus covers around 175 kb of the genome containing several regulatory domains and enhancers (Xiang et al. 2022). *MEIS1* expression has been linked as an oncogene by regulating the proliferation of acute myeloid leukemia (Li et al. 2016; Xiang et al. 2022) and prostate cancer (Johng et al. 2019), migration in pancreatic cancer (von Burstin et al. 2017), and stemness and epithelial-mesenchymal transition (EMT) capability in esophageal squamous cell carcinoma (Mahmoudian et al. 2019; Zargari et al. 2020). Conversely, its expression suppresses colorectal (Li et al. 2022), prostate (Bhanvadia et al. 2018; VanOpstall et al. 2020; Whitlock et al. 2020), lung (Li et al. 2014), and esophageal (Rad et al. 2016) cancers. Previous studies have shown that *MEIS1* is upregulated in gliomas (Berdasco et al. 2009; Vastrad et al. 2017) and its expression influences glioma survival and proliferation (Zha et al. 2014). Recently, it was found that the erythroblast transformation specific (ETS) domain ELF1 TF promotes glioma proliferation, migration, and invasion by increasing Zinc finger protein GFI1 and *MEIS1* interaction (Cheng et al. 2021).

In our study, we focused on the role of *MEIS1* in the development of GSCs. In both *in vitro* and *in vivo* experiments, silencing *MEIS1* expression greatly reduced the proliferation of GSCs. Moreover, *MEIS1* was found to be linked with cell cycle-related genes in integrated transcriptomics analysis. In clinical data, high *MEIS1* expression is related to malignancy and poor outcomes in glioma patients. The findings suggest *MEIS1* as a novel biomarker for glioma patients and a potential therapeutic target for GBM.

## Materials and methods

### Cell culture

Normal (healthy) human astrocyte (NHA) cells were grown in an astrocyte medium (AM; ScienCell Research Laboratories, USA) with 1% astrocyte growth supplement (AGS; ScienCell Research Laboratories, USA), 10% fetal bovine serum (FBS; Gibco, USA), and 1% penicillin/streptomycin (P/S; Welgene, South Korea). Human glioma cell lines (A172, A1207, U87MG, and LN229) were cultured in Dulbecco's modified media (DMEM/F12; Welgene, South Korea), with 1% P/S and 10% FBS. Glioma stem cell lines (GSC11 and GSC23) were received from The University of Texas MD Anderson Cancer Center (Bhat et al. 2013). GSC11 and GSC23 were originally derived from surgical GBM specimens. These lines demonstrate the typical in vitro stem cell traits, such as extensive self-renewal, the capacity to differentiate into neurons and astrocytes, and the capacity to initiate tumors *in vivo* (Yuan et al. 2015). GSC11 and GSC23 were grown in a neurobasal (NBE) media which comprised DMEM/F12 media, 20 ng/ml of epidermal growth factor (EGF; R&D Systems, USA), and basic fibroblast growth factor (bFGF; R&D Systems, USA), 2% B27 (Gibco, USA), and 1% P/S. All cells were maintained at 37°C with 5% CO_2_.

### Lentivirus preparation

Lentiviral vectors generating short-hairpin RNA (shRNA) constructs (shMEIS1_01-TRCN0000434701, shMEIS1_72-TRCN00000159272; Sigma-Aldrich, USA) were used to silence the expression of the *MEIS1* gene. A non-targeting shRNA (shNT; Sigma-Aldrich, USA) with a shRNA-coding sequence that does not target any gene served as a negative control. Plasmid DNA for lentivirus production was packaged in 293FT cells using the CalPhos^TM^ Mammalian Transfection Kit (Clontech Laboratories, Inc., USA). pMD2.G and psPAX2 were used as packaging plasmids. After 72 h of transfection, the lentivirus-containing medium was filtered in a 0.45 µm PES membrane Millex®-HP (Merck Millipore, MA, USA). Finally, a 100-fold concentration was achieved with a Lenti-X™ Concentrator (Clontech Laboratories, Inc., USA). The lentivirus was generated following the manufacturer's directions.

### Cell viability assay

Glioma stem cells (GSC11 and GSC23) were seeded (3×10^5^ cells/well) in a 6-well plate (laminin-coated). After 24 h, cells were infected with lentivirus (shNT, shMEIS1_01, and shMEIS1_72). Cells were incubated for 48 h and then reseeded in a 96-well plate (3000 cells/well; n = 6). After 2-, 4- and 6-days post-transfection, the cell viability was evaluated using the alamarBlue^TM^ cell viability reagent (Invitrogen, USA), as directed by the manufacturer. The absorbance at 590 nm was measured in a Synergy HTX Multi-Mode Reader (BioTek Instruments Inc., USA).

### In vitro limiting dilution assay

The shRNA-transfected GSCs were seeded (96-well plate) at decreasing cell densities (50, 25, 12, 6, 3, and 1 cell(s) per well; *n *= 30). Growth media (10 µl) was added to the wells every 3 days. After 14 days, wells were observed under a light microscope for tumorsphere formation. Positive wells were identified as having tumorspheres diameter bigger than 20μm. The Extreme Limiting Dilution Analysis (ELDA) tool was used to examine the frequency of GSCs’ ability to produce tumorspheres (Hu and Smyth 2009).

### Dataset preparation

The 208 homeobox TF-related genes were identified in the Human Protein Atlas database (https://www.proteinatlas.org/). The GSE4536 dataset was used to assess the mRNA expression profile of homeobox TF-related genes in GSCs under stem cell and differentiated conditions (Lee et al. 2006). Data normalization and transformation (Log2 CPM+1) was performed in the Integrated Differential Expression and Pathway Analysis (iDEP ver.1.1) website (http://bioinformatics.sdstate.edu/idep11/). The Gliovis website (http://gliovis.bioinfo.cnio.es/) was used to retrieve the TCGA_GBMLGG, Gravendeel (GSE16011), REMBRANDT (GSE108474), and Ivy Glioblastoma Atlas Project (Ivy GAP) datasets.

### Bioinformatics analysis

Pearson's R correlation was performed to identify the correlated genes with *MEIS1* expression. Positively moderate to strong correlated genes (R ≥ 0.4; *p* ≤ 0.05) (Akoglu 2018; Schober et al. 2018) to *MEIS1* expression were annotated in the Database for Annotation, Visualization, and Integrated Discovery (DAVID) website (https://david.ncifcrf.gov/), a bioinformatics tool employing gene ontology (GO) and Kyoto Encyclopedia for Genes and Genomes (KEGG) pathways. Gene Set Enrichment Analysis (GSEA) ver. 4.2.2 software was used to identify significantly correlated human MSigDB GO biological processes and KEGG pathway gene sets in patients with high *MEIS1* expression in the TCGA_GBMLGG dataset. The ‘Weighted Gene Co-expression Network Analysis (WGCNA)’ package in R was used to identify gene clusters-modules having strong correlations to *MEIS1* expression and phenotypes. The ‘survival’ and ‘survminer’ package in R were used for univariate and multivariate COX regression analyses. Other data analyses and visualization were performed in the SRplot (https://www.bioinformatics.com.cn/srplot). The TCGA_GBMLGG dataset was used in all analyses.

### Single-cell RNA sequencing analysis

The ‘Seurat’ package in R 4.0.5 was used to analyze the scRNA-seq data of three newly diagnosed GBM samples (GSM5518633, GSM5518634, and GSM5518636) from the GSE182109 dataset (Abdelfattah et al. 2022). Low-quality cells with (<200 total feature RNA; <15% mitochondrial RNA) were removed. The SCTransform integration function was used to merge the data from each dataset. The NormalizedData and ScaleData functions were used to normalize and scale the expression of all genes. The influence of the cell cycle, the total number of counts, and the percentages of genes expressed in mitochondria in each cell were all regressed out of the data after identifying 2000 integration anchors that represent the closest neighbors across datasets. After dimension reduction (npcs = 30) using principal component analysis (PCA), it was further reduced (dims = 1:15) using the uniform manifold approximation and projection (UMAP) method. The FindNeighbors and FindClusters (resolution = 0.1) functions were used to identify the nearest neighbors of single-cell and cluster cells based on their similarity using the Louvain algorithm. Differentially expressed genes in each cluster were identified using the FindAllMarkers function and Wilcoxon rank-sum test (cutoff: min.pct = 0.25 and logfc.threshold = 0.25). Marker genes in each cluster were manually entered into CellMarker2.0 (http://bio-bigdata.hrbmu.edu.cn/CellMarker/index.html) for cell type annotation. Gene scores for Mesenchymal (MES)-like Astrocyte (AC)-like, Oligodendrocyte Progenitor cell (OPC)-like, Neural Progenitor cell (NPC)-like, G1/S, and G2/M (Neftel et al. 2019) in glioma clusters were identified using the AddModuleScore function. Enrichment analysis was performed using the Fast Gene Set Enrichment Analysis (FGSEA) function. The data were visualized using the DimPlot, FeaturePlot, DotPlot, DOHeatmap, and plotEnrichment function.

### RNA-sequencing analysis

GSC11 (3.5×10^5^ cells/well) was seeded in a 6-well plate (laminin-coated). Cells were transfected with either shNT control or shMEIS1_72 lentivirus and then incubated for 48 h. At the end of the incubation period, cells were resuspended in TRIzol® Reagent (Invitrogen; CA, USA) and kept at −80°C. Libraries were constructed using the MGIEasy RNA Directional Library Prep Kit (MGI Tech., Shenzhen, China) following the manufacturer’s procedure. RNA quality control and high-throughput RNA sequencing were performed on the MGISEQ platform (MGI Tech) with 149 bp paired-end reads. Reads were mapped to the hg19 reference genome and expression values were calculated in fragments per kb transcript per million fragments mapped (FPKM). LAS Co., Ltd. (Gimpo, South Korea) performed the RNA purification, library preparation, and sequencing procedures. The new generation of high-throughput sequencing was used to study the differences between the two samples and gene expression in both groups was compared using EdgeR. Significant genes (Log2(FC) cut-off >0.5 and <0.5; *p* ≤ 0.05) were used for functional annotation on the DAVID website. GSEA analysis was also performed to identify the downregulated gene sets after *MEIS1* knockdown.

### Quantitative reverse transcription-PCR (RT-qPCR) analysis

Total RNA was isolated using the GeneAll® RiboEx^TM^ reagent and purified using the GeneAll® Hybrid-R^TM^ kit as per the manufacturer's instructions (GeneALL Biotechnology, South Korea). Total RNA (500 ng) was converted to its complementary DNA (cDNA) using the RevertAid^TM^ First Strand cDNA Synthesis kit (Thermo-Fisher Scientific, USA). RT-qPCR was completed on a qTOWER^3^ Real-Time PCR thermocycler (Analytik-Jena, Germany) using Tli RNaseH Plus TB Green® Premix Ex Taq^TM^ (Takara, South Korea). The relative changes in the gene expression were analyzed using the 2^−ΔΔCt^ method and the results were normalized against the expression level of housekeeping gene 18S. The experiment was independently replicated three times. The primer sequences used in this study are listed in Supplementary Table 1.

### In vivo orthotopic implantation

GSC11 (5 × 10^5^ cells) transfected with shNT or shMEIS1_72 lentivirus were intracranially implanted into the brains of 6- to 8-week-old immunodeficient nude mice at the coordinates of 2.0 mm right and 1.0 mm anterior of the bregma using a Stereotaxic instrument (Harvard Apparatus; MA, USA) (n = 6 mice/group). Mice with a body weight loss of more than 30% were sacrificed. The overall survival was presented using the Kaplan-Meier curve. After one month of GSC injection, one mouse from each shMEIS1_72 and shNT group was euthanized concurrently for brain histological investigation. Chonnam National University's Institutional Animal Care and Use Committee approved all mouse experiments following the Republic of Korea government and institutional standards and laws (CNU IACUC-YB-2021-99).

### In vivo bioluminescence imaging

To maintain nude mice unconscious during imaging procedures, 2% isoflurane was used. Following intraperitoneal injection of D-luciferin (150 mg/kg), mice were photographed in an IVIS® Spectrum *in vivo* imaging system (PerkinElmer; CT, USA) for 10 min. Total flux values were estimated for each mouse using luminous regions of interest (ROI) and are shown in radiance (p/sec/cm^2^/sr).

### Hematoxylin and eosin (H&E) staining

Whole brain samples were obtained from the shNT and shMEIS1 treatment groups. Samples were fixed in 4% paraformaldehyde, followed by paraffin embedding, then sectioned into 4μm thick slices. Brain sections were stained with H&E solutions. Finally, stained sections were examined under a light microscope (DM 750, Leica Microsystems, Wetzlar, Germany) equipped with a digital camera (ICC50E, Leica Microsystems, Wentzler, Germany).

### Statistical analysis

All data analyses were performed in R ver. 4.3.2 and GraphPad Prism version 8.3 (GraphPad Software, San Diego, CA, United States). The statistical significance among groups was determined using a two-tailed t-test and a one-way ANOVA. The data were then compared using Tukey's multiple comparison tests. The mean ± standard error (SE) was used to report all data. The *p-value* ≤ 0.05 was considered statistically significant (*p*<0.05  = *; <0.01 = **; <0.001 = ***; <0.0001 = ****; ns = not significant).

## Results

### MEIS1 is highly expressed in glioma stem cells

**Figure 1.**
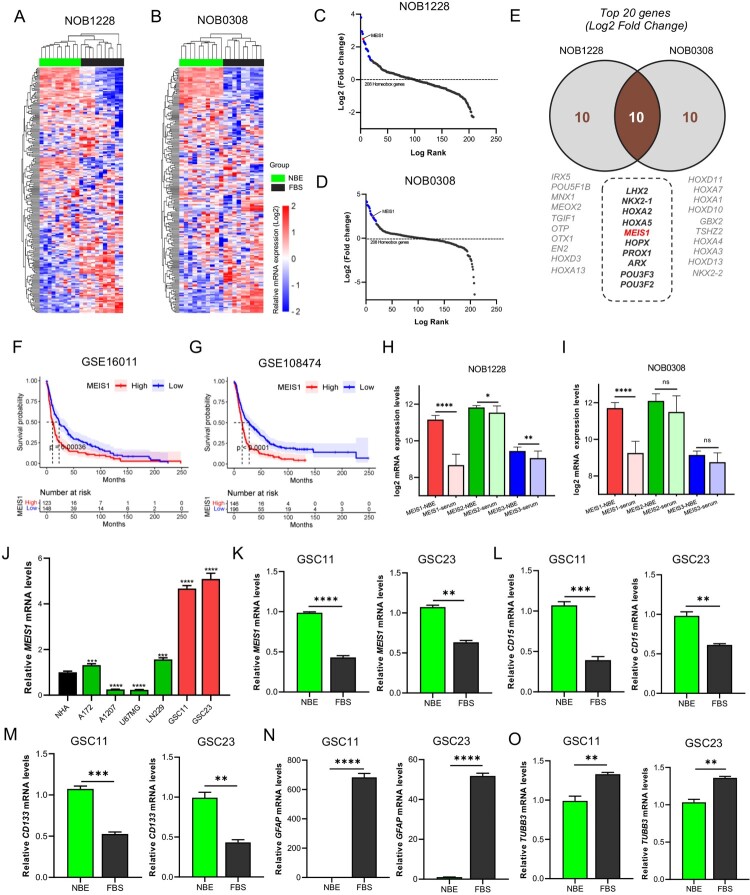
*MEIS1 is highly expressed in GSCs*. (A, B) Cluster heatmap for 208 homeobox-related genes in the GSE4536 dataset. (C, D) Log-rank fold-change for homeobox-related genes in the GSE4536 dataset. (E) The top 20 homeobox-related genes from the NOB1228 and NOB0308 are cell lines displayed in a Ven diagram. (F, G) Kaplan-Meier survival analysis of glioma patients with *MEIS1*^High/Low^ expression in the GSE16011 and GSE10847 datasets. (H, I) mRNA expression levels of MEIS family genes in the GSE4536 dataset; (J) RT-qPCR analysis of the *MEIS1* basal mRNA levels in GBM cell lines (A172, A1207, U87MG, LN229), GSC lines (GSC11, GSC23), and NHA cells. (K-O) RT-qPCR analysis of *MEIS1*, *CD15*, *CD133*, *GFAP*, and *TUBB3* in NBE- and serum-cultured GSC11 and GSC23 cells.

The GSE4536 dataset was used to compare the expression profiles of 208 homeobox TF-related genes in GSCs in stem cell- (NBE) and differentiated- (serum) culture conditions. *MEIS1* is among the highly expressed homeobox genes in both NOB1228 and NOB0308 cell lines under NBE-culture conditions (Figure 1A-D). Filtering out the top 20 upregulated genes, 10 genes (*LHX2, NKX2-1, HOXA2, HOXA5, MEIS1, HOPX, PROX1, ARX, POU3F3, POU3F2*) were found to overlap in the NOB1228 and NOB0308 cell lines (Figure 1E). To validate the clinical significance of these genes, we performed a Kaplan-Meier survival analysis using the GSE16011 and GSE108474 glioma datasets (Figure 1F-G). Results show that high *MEIS1* expression correlates to poor outcomes in glioma patients. We also show that high expression of *HOXA2, HOXA5,* and *ARX* genes influence poor survival, while *NKX2-1* is a good prognostic marker for glioma patients in both datasets (Figure S1). Other overlapped genes showed variable results. The GSE4536 dataset is also used to compare the expression profile of the MEIS family. *MEIS1* is significantly higher in gliomas under NBE-culture conditions, while *MEIS2* and *MEIS3* showed varying expression levels (Figure 1H-I). To validate these findings, we measured the basal mRNA expression levels of *MEIS1* in GSCs (GSC11, GSC23), non-stem cell gliomas (A172, A1207, LN229, U87MG), and NHA cells by RT-qPCR analysis (Figure 1J). We found that *MEIS1* is more highly upregulated in GSCs than in NHA cells and non-stem gliomas. Lastly, we then compared the basal mRNA expression of *MEIS1,* stemness (*CD15, CD133*), and differentiation (*TUBB3, GFAP)* markers in GSC11 and GSC23 under NBE and serum culture conditions. We show that *MEIS1* and stemness markers are significantly upregulated (Figure 1K-M), while differentiation markers are downregulated in GSCs under the NBE-culture condition (Figure 1N-O). These results show that *MEIS1* is highly expressed in GSCs.

### Elevated MEIS1 expression is linked to malignancy and poor survival of glioma patients

**Figure 2.**
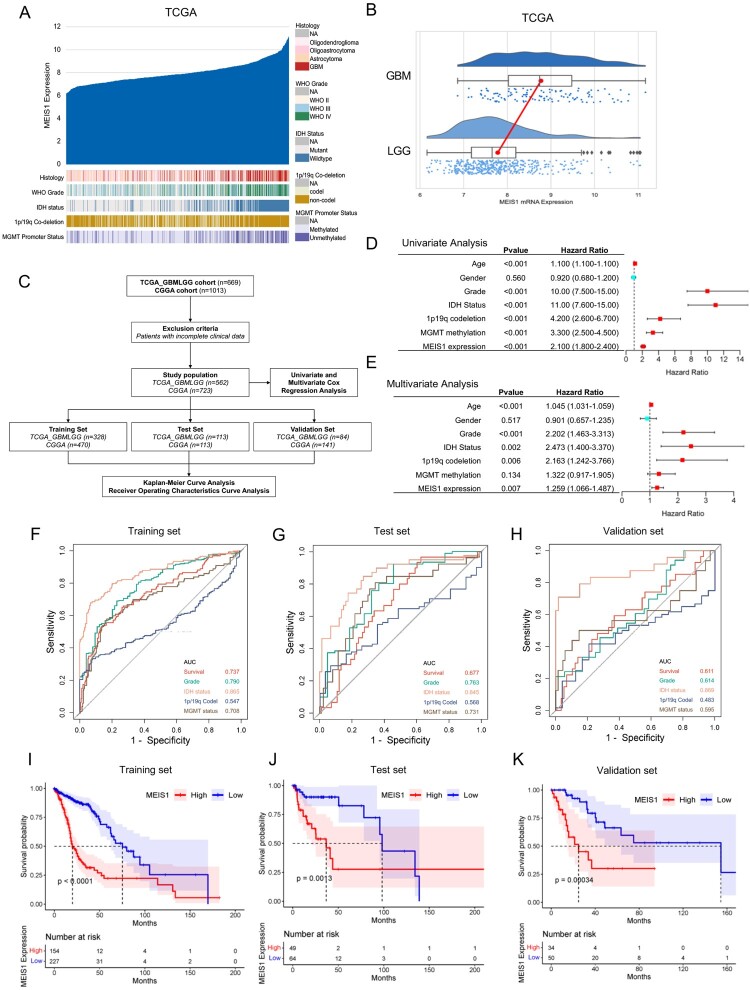
*Elevated expression of MEIS1 is linked to malignancy and poor survival for patients with gliomas.* (A) *MEIS1* mRNA expression and histological features in the TCGA_GBMLGG dataset (n = 669). (B) Raincloud plot of *MEIS1* expression in GBM and LGG patients. (C) Flow chart showing the patient population in the TCGA and CGGA cohorts. (D, E) Univariate and multivariate Cox proportional hazards regression analysis of variables influencing overall patient survival in the TCGA dataset. (F-H) ROC curve and the risk score distribution stratified by *MEIS1* in the TCGA dataset. (I-K) Kaplan-Meier analysis of glioma patients with *MEIS1*^High/Low^ expression in TCGA dataset.

To further understand the clinical relevance of *MEIS1* in glioma*,* we investigated its mRNA expression levels in the TCGA_GBMLGG and CGGA datasets (Figure 2A, S2A). *MEIS1* is highly expressed in GBM (Figure 1A, 1B, S2A, B), and correlated to IDH wildtype, unmethylated MGMT promoter region, and 1p19q non-co-deletion status phenotypes (Figure 2A, S2A). To ascertain the prognostic significance of *MEIS1* expression, we carried out univariate and multivariate, ROC curve, and Kaplan-Meir survival analyses (Figure 2C). Both univariate and multivariate analyses show that high *MEIS1* expression is a predictor for overall survival for glioma patients (Figure 2D-E, S2C-D). Age, gender, grade, IDH, 1p19q codeletion, and MGMT status were also independent predictors for overall patient survival. Meanwhile, we used a ROC curve to analyze the predictive efficacy of *MEIS1* expression (Figure 2F-H, S2E-G). We show that *MEIS1* expression predicts survival well independently of other clinical variables in the training, test, and validation datasets (AUC >0.6). Kaplan-Meier survival analysis shows that high *MEIS1* expression is related to lower patient survival (Figure 2I-K, S2H-J). These findings imply that high MEIS1 expression may be a predictor of prognosis and overall survival of glioma patients.

#### MEIS1 is increased in the GBM classical subtype and enriched in the cellular tumor region

**Figure 3.**
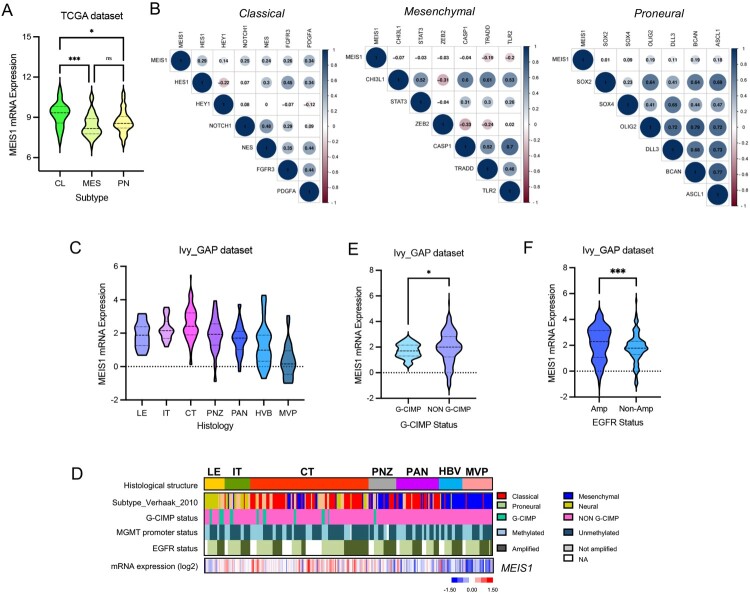
*MEIS1 is increased in the classical GBM subtype and enriched in the cellular tumor region*. (A) *MEIS1* mRNA levels in GBM subtypes from the TCGA dataset (CL - Classical, ME – Mesenchymal, PN – Proneural). (B) Correlation of *MEIS1* expression with CL, MES, and PN markers in the TCGA dataset. (C) *MEIS1* mRNA levels in various GBM regions as described in the IvyGAP dataset. (LE – leading edge; IT – infiltration tumor; CT – cellular tumor; PNZ – perinecrotic zone; PAN – pseudopalisading cells around necrosis; HBV – hyperplastic blood vessels; MVP – microvascular proliferation). (D) Heatmap of *MEIS1* expression and phenotypes in the Ivy GAP dataset. (E, F) *MEIS1* expression based on CIMP and EGFR amplification status in the Ivy GAP dataset.

The TCGA dataset was then used to compare the expression of *MEIS1* in the different GBM subtypes. We show that *MEIS1* is highly expressed in the GBM classical subtype (Figure 3A). To verify this result, we perform correlation analysis in MEIS1 expression and marker genes for each subtype. We show that MEIS1 expression is positively correlated with classical subtype markers (Figure 3B). The Ivy GAP dataset was used to identify the GBM anatomical region that significantly expresses *MEIS1*. We show that *MEIS1* is preferentially elevated in the cellular tumor (CT) region (Figure 3C, D). On the other hand, *MEIS1* is significantly expressed in non-G-CIMP and EGFR-expressing gliomas (Figure 3E, F).

### Bioinformatics analysis reveals the role of MEIS1 expression in gliomas

**Figure 4.**
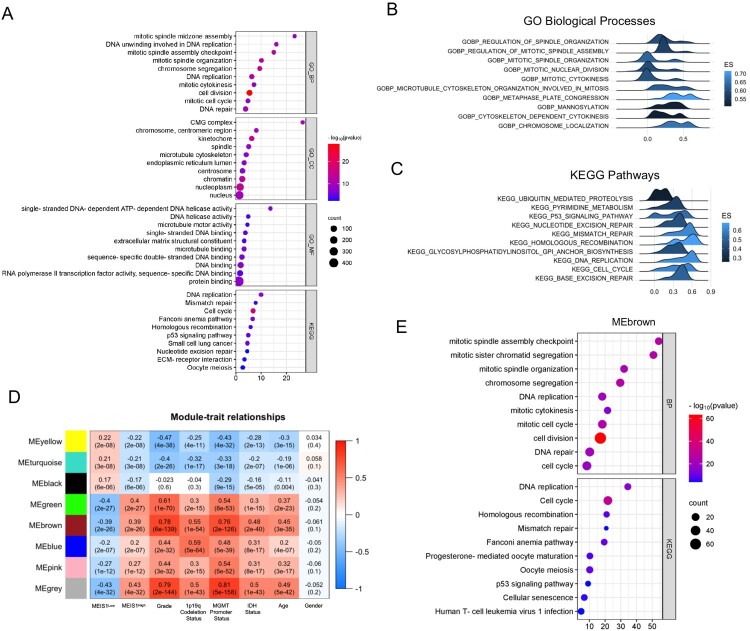
Transcriptomic analysis reveals the role of *MEIS1* in gliomas. (A) DAVID functional annotation of correlated genes with *MEIS1* in TCGA_GBMLGG (Pearson's R>0.4, *p*≤ 0.05). (B, C) GSEA analysis of *MEIS1*^High/Low^ expression in the TCGA_GBMLGG dataset; (D) Relationship between *MEIS1* expression and phenotypes analyzed by WGCNA. (E) DAVID functional annotation of gene hub in the MEbrown module.

Next, we performed Pearson's R correlation analysis to identify the positively correlated genes with *MEIS1* expression in the TCGA dataset. Functional annotation of positively correlated genes (Pearson’s R ≥ 0.40; *p-value* <0.05) revealed that *MEIS1* is associated with DNA replication, DNA repair, cell cycle, and mitosis genes (Figure 4A). The GSEA analysis corroborates these findings (Figure 4B, C). We also performed WGCNA analysis to further elucidate the biological roles of *MEIS1* in gliomas. Interestingly, the gene hub in the MEbrown module ((R = 0.39; *p*<0.0001) validates our findings in the correlation and GSEA analyses (Figure 4D, E, S3). These results show that MEIS1 is crucial in the development and progression of gliomas.

### MEIS1 is enriched in NPC- and OPC-like gliomas according to scRNA-seq analysis

**Figure 5.**
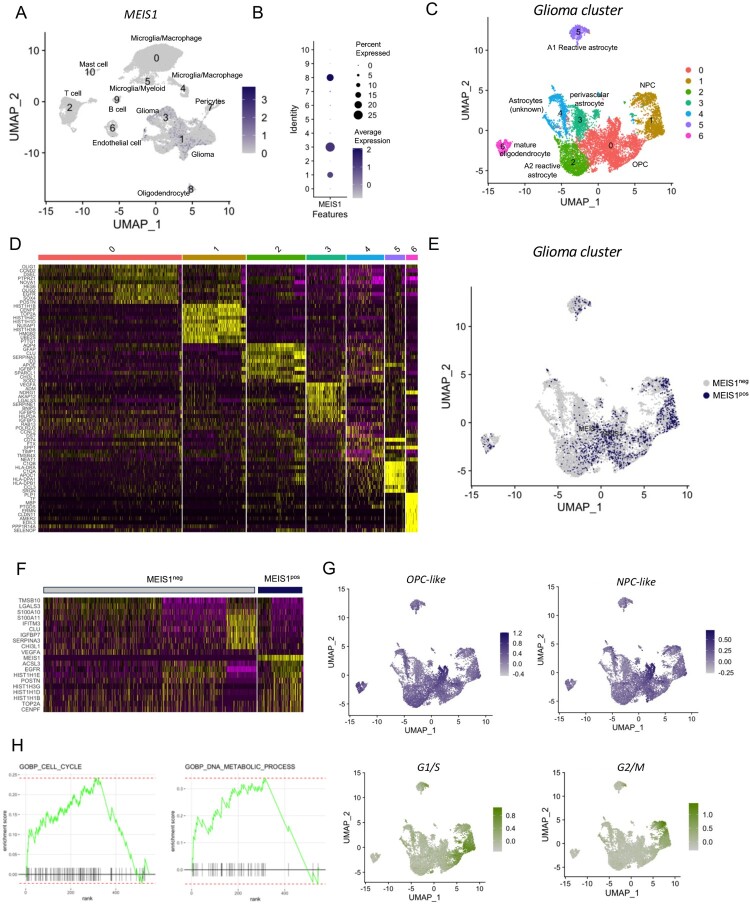
*MEIS1 is enriched in the NPC- and OPC-like gliomas.* (A) FeaturePlot of cell clusters expressing *MEIS1*. (B) DotPlot of *MEIS1* expression in different cell clusters. (C) DimPlot of glioma cell clusters. (D) DOHeatmap of top 10 genes in different glioma cell clusters. (E) FeaturePlot of *MEIS1* expression in different glioma cell clusters. (F) DOHeatmap of top 10 genes enriched in *MEIS1*-positive and *-*negative cells. (G) FeaturePlot of NPC-like, OPC-like, G1/S, and G2/M enrichment in the glioma cell cluster. (H) FGSEA analysis in *MEIS1*^-positive/-negative^ cells.

We also performed a single-cell RNA sequencing analysis of 3 newly diagnosed GBM samples (GSE182109). Different cell clusters were identified including gliomas, stromal, and immune cells (Figures S4A-D). Results show that *MEIS1* is significantly expressed in glioma (Cluster 1, 3) and oligodendrocyte (Cluster 8) cell clusters among the other cell populations (Figure 5A, B). Filtering out the glioma and oligodendrocyte cell population (Figure 5C), we show that *MEIS1* is highly expressed in cell clusters 0 and 1 (Figure 5E). Marker genes for cluster 0 include *OLIG1/2, SOX4*, and *EGFR,* while marker genes for cluster 1 include *CENPF* and *TOP2A* (Figure 5D). Filtering out the *MEIS1*-positive cells, we show that *MEIS1* is co-expressed with *EGFR, POSTN, CENPF*, and *TOP2A* (Figure 5F). These genes are known to be highly expressed in the classical and proneural GBM subtypes. Next, we performed the ‘AddModuleScore’ function in Seurat to calculate module scores for feature expression programs of GBM subtypes (Neftel et al. 2019) in single cells. We show that *MEIS1* is upregulated in the NPC- and OPC-like glioma cells (Figure 5G, S4E-F). Meanwhile, *MEIS1*-expressing expressing cells are located in the highly proliferative region, based on G1/S and G2/M scores (Figure 5G). Lastly, we performed FGSEA analysis to identify the biological processes and functions correlated with *MEIS1* expression. We show that *MEIS1* is positively correlated with as cell cycle, DNA repair, DNA metabolic process, and mitosis, while negatively correlated to apoptosis, focal adhesion, and oxidative phosphorylation (Figure 5H, S4G-J). These findings further validate our results in the bulk-transcriptomics analyses.

### Suppressing MEIS1 inhibits GSC proliferation in vitro and in vivo

**Figure 6.**
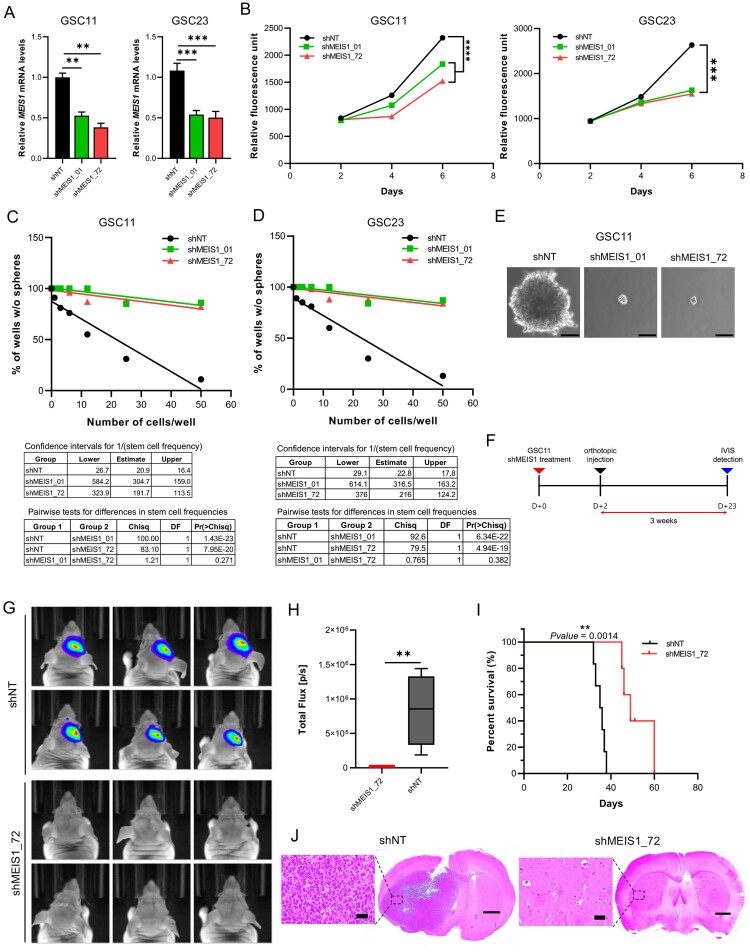
*Suppressing MEIS1 inhibits the GSC proliferation in vitro and in vivo*. RT-qPCR analysis of *MEIS1* in GSC11 and GSC23 cells after shRNA knockdown. (B) Cell viability of GSC11 and GSC23 after *MEIS1* shRNA knockdown. (C) Tumorsphere formation assay in GSC11 and GSC23 after *MEIS1* shRNA knockdown. (D) Representative figure of shNT and shMEIS1_72 transduced GSC11 cells (Scale = 100μm). (E) *In vivo* orthotopic mouse model experimental schedule. (F-G) *In vivo* bioluminescent imaging of nude mice bearing intracranial tumors derived from shNT and shMEIS1_72 knockdown GSC11 cells. (H) Kaplan–Meier curves of nude mice bearing intracranial tumors derived from shNT and shMEIS1_72 knockdown GSC11 cells. (I) Representative H&E-stained brain sections from shNT and shMEIS1 mice groups (Scale = 100 μm).

To elucidate the function of *MEIS1*, we silenced the *MEIS1* expression in GSC using shRNA. RT-qPCR analysis revealed *MEIS1* expression was downregulated by 50% upon shRNA knockdown in both GSC11 and GSC23 cell lines (Figure 6A). The effects of *MEIS1* knockdown on cell viability and tumorsphere formation ability on GSCs were examined *in vitro*. *MEIS1* knockdown significantly reduced cell viability (Figure 6B) and tumorsphere formation in both GSC11 and GSC23 cells (Figure 6C-E). Meanwhile, mRNA levels of stemness and differentiation markers were not influenced by *MEIS1* knockdown (data not shown). The effect of *MEIS1* knockdown on GSC11 tumor formation *in vivo* was further investigated using intracranial injection in an orthotopic animal model (Figure 6F). To enable continuous monitoring of tumor volume, GSC11 was transduced with a constitutive luciferase reporter gene. *In vivo* bioluminescent imaging (Figure 6G, H) and histological analysis (Figure 6J) show that mice implanted with *MEIS1* knockdown GSC11 had much lower tumor growth compared to the shNT control group. Moreover, Kaplan-Meier survival analysis shows that mice implanted with *MEIS1* knockdown GSC11 had significantly greater median survival rates than the shNT group (Figure 6I). These data indicate that *MEIS1* is required for GSC growth.

### Silencing MEIS1 influences the expression of cell cycle-related genes in GSC

**Figure 7.**
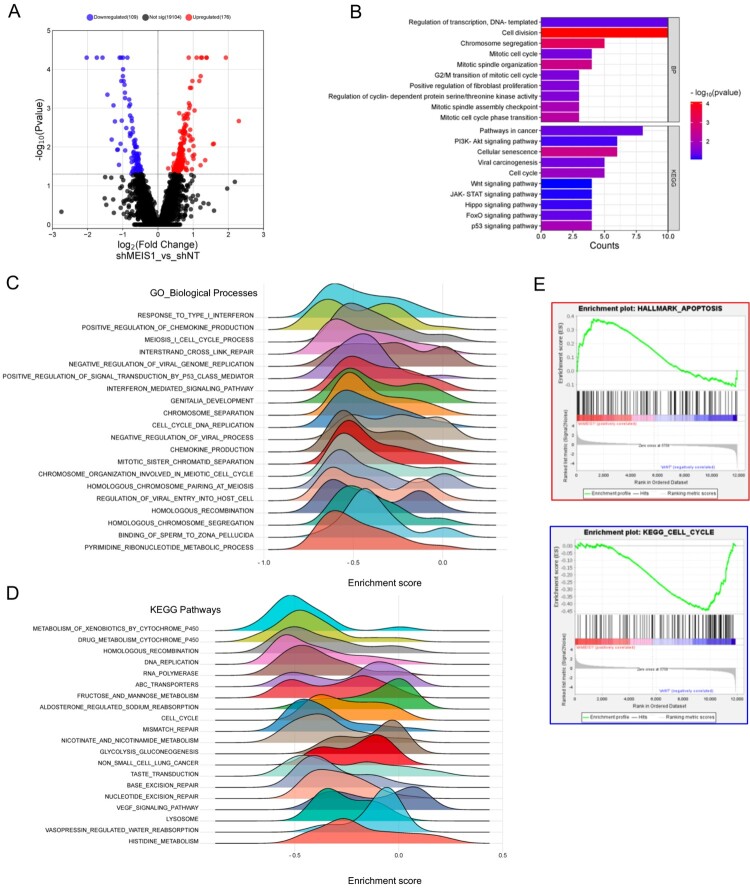
Silencing *MEIS1* downregulates the expression of cell-cycle-related genes. (A) Volcano plot of the DEGs in shNT and shMEIS1 treated GSC11 cells. (B) Functional annotation of genes that are significantly downregulated in shMEIS1-treated GSC11 cells (Log2FC <−0.5; *p* ≤0.05). (C-E) Enrichment plot of downregulated biological processes and pathways in GSC11 cells after *MEIS1* shRNA knockdown.

Finally, we performed RNA-sequencing analysis to identify the differentially expressed genes upon *MEIS1* knockdown in GSC11 cells. We show that there are 109 downregulated and 176 upregulated genes upon *MEIS1* knockdown (Figure 7A). Functional annotation and GSEA analysis revealed that genes linked to the cell cycle, cell division, mitosis, and DNA replication were downregulated in *MEIS1* knockdown cells (Figure 7B-E). Interestingly, we found that apoptosis-related genes were upregulated upon *MEIS1* knockdown. These results show that *MEIS1* is important in the maintenance and proliferation of GSCs.

## Discussion

MEIS1, a TF belonging to the TALE homeobox family, is essential for cell growth and development (Li et al. 2022). In several solid tumors, such as breast, colorectal, human esophageal squamous cell, ovarian, and prostate cancers, *MEIS1* plays an oncogenic role (Blasi and Bruckmann 2021). Moreover, it has been observed that *MEIS1* is upregulated in neuroblastoma and glioma cells, supporting cell survival and proliferation (Berdasco et al. 2009; Zha et al. 2014; Vastrad et al. 2017; Girgin et al. 2020). While *MEIS1* has been shown to play an oncogenic role in glioma and other cancers, its function in GSC is still unclear. *MEIS1* is significantly expressed in GSCs, according to our in-silico and validation experiments, and its expression may have an impact on the aggressive nature of GSCs.

We also found that *HOXA2*, *HOXA5*, and *ARX* are differentially expressed in GSC. *HOXA2* and *HOXA5* are aberrantly expressed in GBM and contribute to poor outcomes in glioma patients (Liu et al. 2020; He et al. 2022). Recently, it was found that *HOXA5* can transcriptionally activate *PTPRZ1* to influence the self-renewal and invasive properties of GSC (He et al. 2022). Meanwhile, the oncogenic role of the *ARX* gene has not been reported to our knowledge, thus further studies are needed. Several studies have shown that the interaction of MEIS and PBX-HOX proteins may act as an oncogene in various cellular settings (Girgin et al. 2020; Jiang et al. 2021). For example, *MEIS1* overexpression and its interaction with *HOXA9* or *PBX* promote proliferation in leukemia cells. Also, MEIS1-HOXB13 and MEIS1-SOX2 promote proliferation in prostate cancer and esophageal squamous cell carcinoma, respectively (Yao et al. 2021). The co-expression and possible interaction of *MEIS1* and its co-factors might influence the aggressive phenotype of GSC, thus more research is needed.

Accordingly, *MEIS1* expression has been noted to be correlated to poor prognosis of various cancers (Schulte and Geerts 2019). Our findings show that MEIS1 is highly expressed in high-grade glioma and was significantly associated with decreased overall survival in glioma datasets. Univariate and multivariate analyses revealed *MEIS1* expression to be an independent prognosticator in gliomas. These findings suggest for the first time that *MEIS1* could be a prognostic biomarker for gliomas.

*MEIS1* is upregulated in the GBM classical subtype. The GBM classical subtype is characterized by amplification of Chr.7, loss of Chr.10, EGFR amplification, inactivation of RB pathway, homozygous deletion of focal 9p21.3, and upregulation of Sonic Hedgehog and Notch signaling pathway genes (Zhang et al. 2020). *Meis1* is reported to upregulate Notch pathway genes (Isogai et al. 2022). Notch plays a role in the maintaining of neural stem cells and controlling cell fate decisions in both the embryonic and adult brain, and it is connected with the aggressive characteristics of GBM (Tulip et al. 2021). On the other hand, *MEIS1* is upregulated in cellular tumors. A recent study shows that the progression of the tumor originates from the tumor core to the leading edge of the tumor through the intermediate state or toward a proliferating state close to the vasculature or necrotic state (Puchalski et al. 2018; Celiku et al. 2019). Further studies are needed to elucidate the role of *MEIS1* in the aggressive characteristics of GBM classical subtype and cellular tumors.

Integrated transcriptomics analysis reveals the role of *MEIS1* in gliomas. We show that *MEIS1* expression is positively correlated with DNA replication, DNA repair, cell cycle progression, mitosis, and cell division genes. Our *in vitro* and *in vivo* experiments show that *MEIS1* expression is linked to increased GSC cell proliferation and progression. Moreover, *MEIS1* knockdown influenced cell cycle-related pathways in GSC cells. In neuroblastoma, *MEIS2* is important for preserving the expression of late cell-cycle genes, including those required for M-phase progression, G2-M checkpoint regulation, and DNA replication (Zha et al. 2014). Recently, it was found that *MEIS1* is activated by TF-*ELF1*, then regulates the *GFI1/FBW7* axis to promote gliomagenesis (Cheng et al. 2021). *MEIS1* expression is increased in malignant peripheral nerve sheath tumors, where it inhibits the p27 cell cycle suppressor through TF inhibitor of DNA binding 1 (*ID1*), thereby promoting cell proliferation and sustaining cell survival (Patel et al. 2016). Additional study is necessary to understand the mechanism by which *MEIS1* regulates the cell cycle to promote GSC progression.

We also employed single-cell transcriptomics analysis to elucidate the importance of *MEIS1* in the tumor microenvironment (TME). The results reveal that *MEIS1* is preferentially expressed in gliomas, particularly in the NPC- and OPC-like glioma clusters which corresponds to classical and proneural GBM subtypes. Moreover, *MEIS1* is enriched in the highly proliferative region of the cell cluster based on the G1/S and G2/M scores. FGSEA analysis further proves that *MEIS1* co-expressed with cell cycle, DNA repair, and DNA metabolic process genes. A study revealed a high correlation between MEIS1 expression and the majority of immune cells in various cancers (Li et al. 2023). For example, the FTO/RP5-991G20.1/hsa-miR-1976/MEIS1 regulatory axis is necessary for regulating the infiltration of immune cells and prognosis in ovarian cancer patients (Chen et al. 2023). Additionally, in early-stage ovarian cancer, MEIS1 contributes to CD8+ T-lymphocyte infiltration and stimulates the synthesis of chemokines (Karapetsas et al. 2018). A more in-depth understanding of how *MEIS1* impacts the TME, particularly in GBM, is required in the future.

The oncogenic role of *MEIS1* is affected by multiple factors, and the complex role of *MEIS1* in proliferation may largely depend on the TME (Meng et al. 2021). MEIS-HOX proteins interaction may transactivate HIFs to promote aberrant cell growth in the hypoxic TME, hence contributing to the Warburg effect (Girgin et al. 2020). Conversely, *MEIS1* deletion decreases *HIF1A* and *HIF2A* expression in HSCs, leading to mitochondrial metabolism increased reactive oxygen species generation, and apoptosis (Kocabas et al. 2012). Our scRNA sequencing analysis shows that MEIS1 expression is negatively correlated with apoptosis and oxidative phosphorylation genes. Meanwhile, RNA sequencing analysis shows knockdown GSCs show that apoptosis genes are upregulated. Studies show that a decrease in *MEIS1* in perinatal cardiomyocytes promotes the maturation of oxidative phosphorylation (Lindgren et al. 2019). Also, MEIS1-HOXA9 interaction prevents apoptosis and protects cells against the effect of apoptosis-inducing factors, leading to leukemogenesis (Wermuth and Buchberg 2005). A recent study shows that inhibition of *MEIS1* in primary leukemia cells reduced cell viability and stem cell property by inducing apoptosis (Meriç et al. 2024). Hence, further investigation is needed to elucidate the role of *MEIS1* in oxidative phosphorylation and apoptosis, particularly in GSC.

A study shows that *MEIS1* inhibition can enhance apoptosis and hampered self-renewal in adult HSCs via downregulating *HIF1A* and *HIF2A* (Jiang et al. 2021). Accordingly, stabilizing *HIF2A* increases the expression of stem cell factors such as *OCT4*, *SOX2*, and *NANOG*. Conversely, *HIF2A* knockdown inhibits GSC self-renewal and tumor formation *in vitro* and *in vivo* (Boyd et al. 2021). MEIS1-SOX2 interaction maintains the stem cell status and cell proliferation in esophageal squamous cell carcinoma cells (Zargari et al. 2020). In our study, there was no significant effect in the expression of stemness markers after *MEIS1* knockdown in GSCs. Therefore, more research is required to understand the mechanism of how *MEIS1* expression could influence the stemness or self-renewal property of GSCs.

We also observed the downregulation of the ABC transporters pathway as *MEIS1* is inhibited. The RNA sequencing data revealed that *ABCC6* was substantially downregulated gene upon MEIS1 knockdown. Recent studies have consistently indicated that the *ABCC6* gene is a significant factor in developing resistance to pharmacological treatments. In breast cancer cells, the overexpression of the *ABCC6P1* pseudogene enhances drug resistance, likely through the activation of its ancestral gene, *ABCC6* (Hashemi and Golalipour 2022). In another study, the efficacy of Subtrifloralactone G extracted from *Deprea subtriflora* in targeting *ABCC6* was demonstrated, highlighting its promise as a therapeutic agent for drug-resistant triple-negative breast cancer (Pandya et al. 2020). *ABCC6* expression, enhanced by *ID4*-mediated *SOX2* induction, plays a pivotal role in imparting chemoresistance to GSCs, highlighting a key mechanism in glioma cell drug resistance (Jeon et al. 2011). However, there is yet to be research demonstrating that *MEIS1* regulates drug resistance through *ABCC6*. Future investigations into the role of GSCs in mediating drug resistance via *MEIS1-ABCC6* interactions might offer novel therapeutic targets for GBM treatment.

In conclusion, *MEIS1* is highly expressed in GSCs, and its expression influences GSC proliferation and development. We propose that *MEIS1* is a novel biomarker for GBM and a potential therapeutic target. Therefore, there is need for a continued efforts to study the role of *MEIS1* in aggressive GSC behavior, as this may represent potentially novel targets for future therapies. Specifically, *MEIS1* direct targeting in GSC may open up new possibilities for inhibiting its molecular interactions with other oncogenic transcription factors, which in turn enables the development of novel treatments such as small peptide inhibitors.

## Author contributions

Conceptualization: H-JK, S-HK; Acquisition of data: H-JK, DRB; Analysis and interpretation of data: H-JK, DRB, Y-JJ, SSL, SB, S-HK; Writing and editing of the manuscript: DRB, H-JK, Y-JJ, SSL, SB, S-HK; Funding acquisition: SB, S-HK; Study supervision: S-HK.

## Supplementary Material

Supplemental Material

## References

[CIT0001] Abdelfattah N, Kumar P, Wang C, Leu J-S, Flynn WF, Gao R, Baskin DS, Pichumani K, Ijare OB, Wood SL, et al. 2022. Single-cell analysis of human glioma and immune cells identifies S100A4 as an immunotherapy target. Nat Commun. 13(1):767. doi:10.1038/s41467-022-28372-y. 35140215 PMC8828877

[CIT0002] Akoglu H. 2018. User’s guide to correlation coefficients. Turk J Emerg Med. 18(3):91–93. doi:10.1016/j.tjem.2018.08.001. 30191186 PMC6107969

[CIT0003] Alves ALV, Gomes INF, Carloni AC, Rosa MN, da Silva LS, Evangelista AF, Reis RM, Silva VAO. 2021. Role of glioblastoma stem cells in cancer therapeutic resistance: a perspective on antineoplastic agents from natural sources and chemical derivatives. Stem Cell Res Ther [Internet]. 12(1):206. doi:10.1186/s13287-021-02231-x. 33762015 PMC7992331

[CIT0004] Arunachalam E, Rogers W, Simpson GR, Möller-Levet C, Bolton G, Ismael M, Smith C, Keegen K, Bagwan I, Brend T, et al. 2022. HOX and PBX gene dysregulation as a therapeutic target in glioblastoma multiforme. BMC Cancer. 22(1). doi:10.1186/s12885-022-09466-8. PMC9006463 35418059

[CIT0005] Berdasco M, Ropero S, Setien F, Fraga MF, Lapunzina P, Losson R, Alaminos M, Cheung N-K, Rahman N, Esteller M. 2009. Epigenetic inactivation of the Sotos overgrowth syndrome gene histone methyltransferase NSD1 in human neuroblastoma and glioma. Proc Natl Acad Sci USA. 106(51):21830–21835. doi:10.1073/pnas.0906831106. 20018718 PMC2793312

[CIT0006] Bhanvadia RR, VanOpstall C, Brechka H, Barashi NS, Gillard M, McAuley EM, Vasquez JM, Paner G, Chan W-C, Andrade J, et al. 2018. MEIS1 and MEIS2 expression and prostate cancer progression: a role for HOXB13 binding partners in metastatic disease. Clin Cancer Res [Internet]. 24(15):3668–3680. doi:10.1158/1078-0432.CCR-17-3673. 29716922 PMC6082699

[CIT0007] Bhat KPL, Balasubramaniyan V, Vaillant B, Ezhilarasan R, Hummelink K, Hollingsworth F, Wani K, Heathcock L, James JD, Goodman LD, et al. 2013. Mesenchymal differentiation mediated by NF-κB promotes radiation resistance in glioblastoma. Cancer Cell. 24(3):331–346. doi:10.1016/j.ccr.2013.08.001. 23993863 PMC3817560

[CIT0008] Blasi F, Bruckmann C. 2021. MEIS1 in hematopoiesis and cancer. How MEIS1-PBX interaction can be used in therapy. J Dev Biol. 9(4):44. doi:10.3390/jdb9040044. 34698191 PMC8544432

[CIT0009] Boyd NH, Tran AN, Bernstock JD, Etminan T, Jones AB, Gillespie GY, Friedman GK, Hjelmeland AB. 2021. Glioma stem cells and their roles within the hypoxic tumor microenvironment. Theranostics. 11(2):665–683. doi:10.7150/thno.41692. 33391498 PMC7738846

[CIT0010] Brotto DB, Siena ÁDD, de Barros II, Carvalho S da CES, Muys BR, Goedert L, Cardoso C, Plaça JR, Ramão A, Squire JA, et al. 2020. Contributions of HOX genes to cancer hallmarks: enrichment pathway analysis and review. Tumour Biol [Internet]. 42(5):1010428320918050. doi:10.1177/1010428320918050. 32456563

[CIT0011] Celiku O, Gilbert MR, Lavi O. 2019. Computational modeling demonstrates that glioblastoma cells can survive spatial environmental challenges through exploratory adaptation. Nat Commun. 10(1):5704. doi:10.1038/s41467-019-13726-w. 31836713 PMC6911112

[CIT0012] Chen L, Gao W, Lin L, Sha C, Li T, Chen Q, Wei H, Yang M, Xing J, Zhang M, et al. 2023. A methylation- and immune-related lncRNA signature to predict ovarian cancer outcome and uncover mechanisms of chemoresistance. J Ovarian Res. 16(1):186. doi:10.1186/s13048-023-01260-9. 37674251 PMC10483746

[CIT0013] Cheng M, Zeng Y, Zhang T, Xu M, Li Z, Wu Y. 2021. Transcription factor ELF1 activates MEIS1 transcription and then regulates the GFI1/FBW7 axis to promote the development of glioma. Mol Ther Nucleic Acids. 23:418–430. doi:10.1016/j.omtn.2020.10.015. 33473327 PMC7787950

[CIT0014] Davis M. 2016. Glioblastoma: overview of disease and treatment. Clin J Oncol Nurs [Internet]. 20(5):S2–S8. doi:10.1188/16.CJON.S1.2-8. PMC5123811 27668386

[CIT0015] Feng Y, Zhang T, Wang Y, Xie M, Ji X, Luo X, Huang W, Xia L. 2021. Homeobox genes in cancers: from carcinogenesis to recent therapeutic intervention. Front Oncol [Internet]. 11. doi:10.3389/fonc.2021.770428. PMC8551923 34722321

[CIT0016] Girgin B, Karadağ-Alpaslan M, Kocabaş F. 2020. Oncogenic and tumor suppressor function of meis and associated factors. Turk J Biol. 44(6):328–355. doi:10.3906/biy-2006-25. 33402862 PMC7759197

[CIT0017] Gonçalves CS, Le Boiteux E, Arnaud P, Costa BM. 2020. HOX gene cluster (de)regulation in brain: from neurodevelopment to malignant glial tumours. Cell Mol Life Sci [Internet]. 77(19):3797–3821. doi:10.1007/s00018-020-03508-9. 32239260 PMC11105007

[CIT0018] Hashemi M, Golalipour M. 2022. ABCC6P1 pseudogene induces ABCC6 upregulation and multidrug resistance in breast cancer. Mol Biol Rep. 49(10):9633–9639. doi:10.1007/s11033-022-07872-6. 36030475

[CIT0019] He Z-C, Liu Q, Yang K-D, Chen C, Zhang X-N, Wang W-Y, Zeng H, Wang B, Liu Y-Q, Luo M, et al. 2022. HOXA5 is amplified in glioblastoma stem cells and promotes tumor progression by transcriptionally activating PTPRZ1. Cancer Lett [Internet]. 533:215605. doi:10.1016/j.canlet.2022.215605. 35219772

[CIT0020] Hu Y, Smyth GK. 2009. ELDA: extreme limiting dilution analysis for comparing depleted and enriched populations in stem cell and other assays. J Immunol Methods. 347(1–2):70–78. doi:10.1016/j.jim.2009.06.008. 19567251

[CIT0021] Isogai E, Okumura K, Saito M, Tokunaga Y, Wakabayashi Y. 2022. Meis1 plays a roles in cortical development through regulation of cellular proliferative capacity in the embryonic cerebrum. Biomed Res [Internet]. 43(3):91–97. doi:10.2220/biomedres.43.91. 35718449

[CIT0022] Jeon H-M, Sohn Y-W, Oh S-Y, Kim S-H, Beck S, Kim S, Kim H. 2011. ID4 imparts Chemoresistance and cancer stemness to glioma cells by derepressing miR-9*–Mediated suppression of SOX2. Cancer Res. 71(9):3410–3421. doi:10.1158/0008-5472.CAN-10-3340. 21531766

[CIT0023] Jiang M, Xu S, Bai M, Zhang A. 2021. The emerging role of MEIS1 in cell proliferation and differentiation. Am J Physiol-Cell Physiol. 320(3):C264–C269. doi:10.1152/ajpcell.00422.2020. 33296285

[CIT0024] Johng D, Torga G, Ewing CM, Norris JK, McDonnell JD, Isaacs DP, B W. 2019. HOXB13 interaction with MEIS1 modifies proliferation and gene expression in prostate cancer. Prostate [Internet]. 79(4):414–424. doi:10.1002/pros.23747. 30560549

[CIT0025] Karapetsas A, Tokamani M, Evangelou C, Sandaltzopoulos R. 2018. The homeodomain transcription factor MEIS1 triggers chemokine expression and is involved in CD8+ T-lymphocyte infiltration in early stage ovarian cancer. Mol Carcinog. 57(9):1251–1263. doi:10.1002/mc.22840. 29802738

[CIT0026] Kocabas F, Zheng J, Thet S, Copeland NG, Jenkins NA, DeBerardinis RJ, Zhang C, Sadek HA. 2012. Meis1 regulates the metabolic phenotype and oxidant defense of hematopoietic stem cells. Blood. 120(25):4963–4972. doi:10.1182/blood-2012-05-432260. 22995899 PMC3525021

[CIT0027] Lee J, Kotliarova S, Kotliarov Y, Li A, Su Q, Donin NM, Pastorino S, Purow BW, Christopher N, Zhang W, et al. 2006. Tumor stem cells derived from glioblastomas cultured in bFGF and EGF more closely mirror the phenotype and genotype of primary tumors than do serum-cultured cell lines. Cancer Cell. 9(5):391–403. doi:10.1016/j.ccr.2006.03.030. 16697959

[CIT0028] Li H, Tang Y, Hua L, Wang Z, Du G, Wang S, Lu S, Li W. 2023. A systematic pan-Cancer analysis of MEIS1 in human tumors as prognostic biomarker and immunotherapy target. J Clin Med. 12(4):1646. doi:10.3390/jcm12041646. 36836180 PMC9964192

[CIT0029] Li W, Huang K, Guo H, Cui G. 2014. Meis1 regulates proliferation of non-small-cell lung cancer cells. J Thorac Dis. 6(6):850–855. doi:10.3978/j.issn.2072-1439.2014.06.03. 24977012 PMC4073365

[CIT0030] Li Y, Gan Y, Liu J, Li J, Zhou Z, Tian R, Sun R, Liu J, Xiao Q, Li Y, et al. 2022. Downregulation of MEIS1 mediated by ELFN1-AS1/EZH2/DNMT3a axis promotes tumorigenesis and oxaliplatin resistance in colorectal cancer. Signal Transduct Target Ther. 7(1). doi:10.1038/s41392-022-00902-6. PMC8964798 35351858

[CIT0031] Li Z, Chen P, Su R, Hu C, Li Y, Elkahloun AG, Zuo Z, Gurbuxani S, Arnovitz S, Weng H, et al. 2016. PBX3 and MEIS1 cooperate in hematopoietic cells to drive acute myeloid leukemias characterized by a core transcriptome of the MLL-rearranged disease. Cancer Res. 76(3):619–629. doi:10.1158/0008-5472.CAN-15-1566. 26747896 PMC4810030

[CIT0032] Lindgren IM, Drake RR, Chattergoon NN, Thornburg KL. 2019. Down-regulation of MEIS1 promotes the maturation of oxidative phosphorylation in perinatal cardiomyocytes. FASEB J. 33(6):7417–7426. doi:10.1096/fj.201801330RR. 30884246 PMC6529342

[CIT0033] Liu Z, Shen F, Wang H, Li A, Wang J, Du L, Liu B, Zhang B, Lian X, Pang B, et al. 2020. Abnormally high expression of HOXA2 as an independent factor for poor prognosis in glioma patients. Cell Cycle. 19(13):1632–1640. doi:10.1080/15384101.2020.1762038. 32436804 PMC7469623

[CIT0034] Mahmoudian RA, Bahadori B, Rad A, Abbaszadegan MR, Forghanifard MM. 2019. MEIS1 knockdown may promote differentiation of esophageal squamous carcinoma cell line KYSE-30. Mol Genet Genomic Med. 7(7). doi:10.1002/mgg3.746. PMC6625128 31090196

[CIT0035] Mallo M, Wellik DM, Deschamps J. 2010. Hox genes and regional patterning of the vertebrate body plan. Dev Biol [Internet]. 344(1):7–15. doi:10.1016/j.ydbio.2010.04.024. 20435029 PMC2909379

[CIT0036] Meng L, Tian Z, Wang J, Liu X, Zhang W, Hu M, Wang M, Zhang Y. 2021. Effect of myeloid ecotropic viral integration site (MEIS) family genes on tumor microenvironment remodeling and its potential therapeutic effect. Transl Androl Urol. 10(2):594–608. doi:10.21037/tau-20-1163. 33718062 PMC7947450

[CIT0037] Meriç N, Albayrak E, Gülbaş Z, Kocabaş F. 2024. MEIS inhibitors reduce the viability of primary leukemia cells and stem cells by inducing apoptosis. Leuk Lymphoma. 65(2):187–198. doi:10.1080/10428194.2023.2275532. 37902585

[CIT0038] Neftel C, Laffy J, Filbin MG, Hara T, Shore ME, Rahme GJ, Richman AR, Silverbush D, Shaw ML, Hebert CM, et al. 2019. An integrative model of cellular states, plasticity, and genetics for glioblastoma. Cell. 178(4):835–849.e21. doi:10.1016/j.cell.2019.06.024. 31327527 PMC6703186

[CIT0039] Pandya H, Patel CN, Bhavsar M, Pandya PN, Patel SK, Rawal RM. 2020. Analyzing the role of phytochemicals in targeting drug transporter protein ABCC6 using molecular docking and molecular dynamics simulations. Int J Pharm Sci Drug Res. 275–281. doi:10.25004/IJPSDR.2020.120310.

[CIT0040] Park DM, Rich JN. 2009. Biology of glioma cancer stem cells. Mol Cells. 28(1):7–12. doi:10.1007/s10059-009-0111-2. 19655094

[CIT0041] Patel AV, Chaney KE, Choi K, Largaespada DA, Kumar AR, Ratner N. 2016. An ShRNA screen identifies MEIS1 as a driver of malignant peripheral nerve sheath tumors. EBioMedicine. 9:110–119. doi:10.1016/j.ebiom.2016.06.007. 27333032 PMC4972548

[CIT0042] Prager BC, Bhargava S, Mahadev V, Hubert CG, Rich JN. 2020. Glioblastoma stem cells: driving resilience through Chaos. Trends Cancer [Internet]. 6(3):223–235. doi:10.1016/j.trecan.2020.01.009. 32101725 PMC8779821

[CIT0043] Puchalski RB, Shah N, Miller J, Dalley R, Nomura SR, Yoon J-G, Smith KA, Lankerovich M, Bertagnolli D, Bickley K, et al. 2018. An anatomic transcriptional atlas of human glioblastoma. Science (1979). 360(6389):660–663. doi:10.1126/science.aaf2666. PMC6414061 29748285

[CIT0044] Rad A, Farshchian M, Forghanifard MM, Matin MM, Bahrami AR, Geerts D, A’rabi A, Memar B, Abbaszadegan MR. 2016. Predicting the molecular role of MEIS1 in esophageal squamous cell carcinoma. Tumor Biology [Internet]. 37(2):1715–1725. doi:10.1007/s13277-015-3780-9. 26314854

[CIT0045] Roh J, Im M, Kang J, Youn B, Kim W. 2023. Long non-coding RNA in glioma: novel genetic players in temozolomide resistance. Anim Cells Syst (Seoul). 27(1):19–28. doi:10.1080/19768354.2023.2175497. 36819921 PMC9937017

[CIT0046] Schober P, Boer C, Schwarte LA. 2018. Correlation Coefficients: Appropriate Use and Interpretation. [place unknown]. doi:10.1213/ANE.0000000000002864. 29481436

[CIT0047] Schulte D, Geerts D. 2019. MEIS transcription factors in development and disease. Development (Cambridge). 146(16). doi:10.1242/dev.174706. 31416930

[CIT0048] Suvà ML, Tirosh I. 2020. The glioma stem cell model in the era of single-cell genomics. Cancer Cell [Internet]. 37(5):630–636. doi:10.1016/j.ccell.2020.04.001. 32396858

[CIT0049] Taylor OG, Brzozowski JS, Skelding KA. 2019. Glioblastoma Multiforme: an overview of emerging therapeutic targets. Front Oncol [Internet]. 9(SEP). doi:10.3389/fonc.2019.00963. PMC6775189 31616641

[CIT0050] Tulip IJ, Kim S-O, Kim E-J, Kim J, Lee JY, Kim H, Kim S-C. 2021. Combined inhibition of STAT and notch signalling effectively suppresses tumourigenesis by inducing apoptosis and inhibiting proliferation, migration and invasion in glioblastoma cells. Anim Cells Syst (Seoul). 25(3):161–170. doi:10.1080/19768354.2021.1942983. 34262659 PMC8253205

[CIT0051] VanOpstall C, Perike S, Brechka H, Gillard M, Lamperis S, Zhu B, Brown R, Bhanvadia R, Vander Griend DJ. 2020. MEIS-mediated suppression of human prostate cancer growth and metastasis through HOXB13-dependent regulation of proteoglycans. Elife [Internet]. 9:1–64. doi:10.7554/eLife.53600. PMC7371429 32553107

[CIT0052] Vastrad B, Vastrad C, Godavarthi A, Chandrashekar R. 2017. Molecular mechanisms underlying gliomas and glioblastoma pathogenesis revealed by bioinformatics analysis of microarray data. Med Oncol. 34(11):182. doi:10.1007/s12032-017-1043-x. 28952134

[CIT0053] von Burstin J, Bachhuber F, Paul M, Schmid RM, Rustgi AK. 2017. The TALE homeodomain transcription factor MEIS1 activates the pro-metastatic melanoma cell adhesion molecule Mcam to promote migration of pancreatic cancer cells. Mol Carcinog [Internet]. 56(3):936–944. doi:10.1002/mc.22547. 27583552

[CIT0054] Wermuth PJ, Buchberg AM. 2005. Meis1-mediated apoptosis is caspase dependent and can be suppressed by coexpression of HoxA9 in murine and human cell lines. Blood. 105(3):1222–1230. doi:10.1182/blood-2004-03-0802. 15479723

[CIT0055] Whitlock NC, Trostel SY, Wilkinson S, Terrigino NT, Hennigan ST, Lake R, Carrabba NV, Atway R, Walton ED, Gryder BE, et al. 2020. MEIS1 down-regulation by MYC mediates prostate cancer development through elevated HOXB13 expression and AR activity. Oncogene [Internet]. 39(34):5663–5674. doi:10.1038/s41388-020-01389-7. 32681068 PMC7441006

[CIT0056] Wu W, Klockow JL, Zhang M, Lafortune F, Chang E, Jin L, Wu Y, Daldrup-Link HE. 2021. Glioblastoma multiforme (GBM): An overview of current therapies and mechanisms of resistance. Pharmacol Res [Internet]. 171:105780. doi:10.1016/j.phrs.2021.105780. 34302977 PMC8384724

[CIT0057] Xiang P, Yang X, Escano L, Dhillon I, Schneider E, Clemans-Gibbon J, Wei W, Wong J, Wang SX, Tam D, et al. 2022. Elucidating the importance and regulation of key enhancers for human MEIS1 expression. Leukemia. 36(8):1980–1989. doi:10.1038/s41375-022-01602-4. 35624144 PMC9343249

[CIT0058] Yao M, Gu Y, Yang Z, Zhong K, Chen Z. 2021. MEIS1 and its potential as a cancer therapeutic target (Review). Int J Mol Med [Internet]. 48(3). doi:10.3892/ijmm.2021.5014. PMC8354308 34318904

[CIT0059] Yuan S, Lu Y, Yang J, Chen G, Kim S, Feng L, Ogasawara M, Hammoudi N, Lu W, Zhang H, et al. 2015. Metabolic activation of mitochondria in glioma stem cells promotes cancer development through a reactive oxygen species-mediated mechanism. Stem Cell Res Ther. 6(1):198. doi:10.1186/s13287-015-0174-2. 26472041 PMC4606508

[CIT0060] Zargari S, Negahban Khameneh S, Rad A, Forghanifard MM. 2020. MEIS1 promotes expression of stem cell markers in esophageal squamous cell carcinoma. BMC Cancer. 20(1). doi:10.1186/s12885-020-07307-0. PMC7441725 32819319

[CIT0061] Zha Y, Xia Y, Ding J, Choi JH, Yang L, Dong Z, Yan C, Huang S, Ding HF. 2014. MEIS2 is essential for neuroblastoma cell survival and proliferation by transcriptional control of M-phase progression. Cell Death Dis. 5(9). doi:10.1038/cddis.2014.370. PMC4540202 25210800

[CIT0062] Zhang P, Xia Q, Liu L, Li S, Dong L. 2020. Current opinion on molecular characterization for GBM classification in guiding clinical diagnosis, prognosis, and therapy. Front Mol Biosci. 7. doi:10.3389/fmolb.2020.562798. PMC7506064 33102518

