# Supplementary figures and images for: The impact of MEIS1 TALE homeodomain transcription factor knockdown on glioma stem cell growth
Source: Anim Cells Syst (Seoul). 2024 Mar 13;28(1):93–109. doi: 10.1080/19768354.2024.2327340 (PMC10939110; doi:10.1080/19768354.2024.2327340)

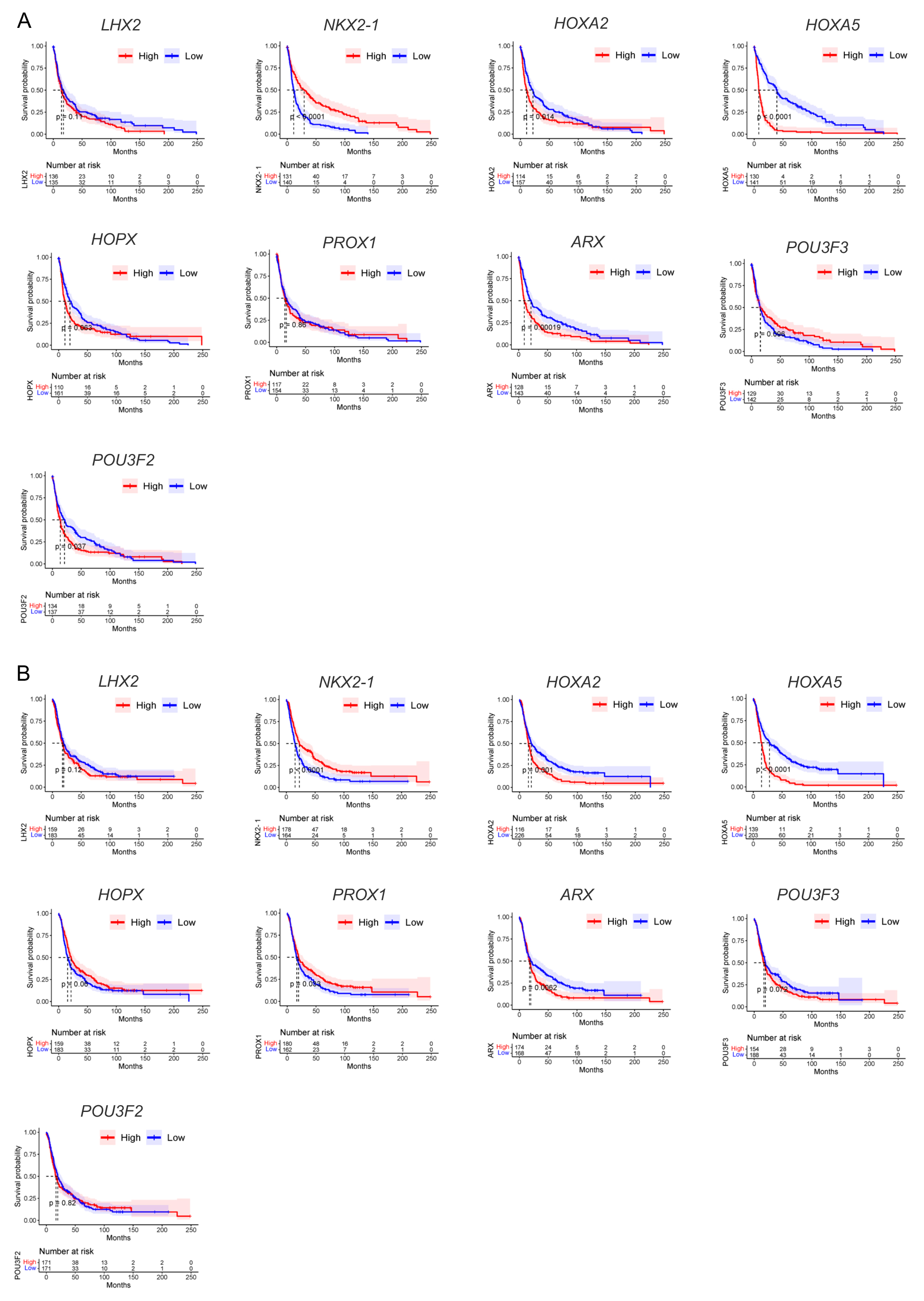

Supplement: Supplemental Material [file TACS_A_2327340_SM4634.zip › FigureS1.jpg]

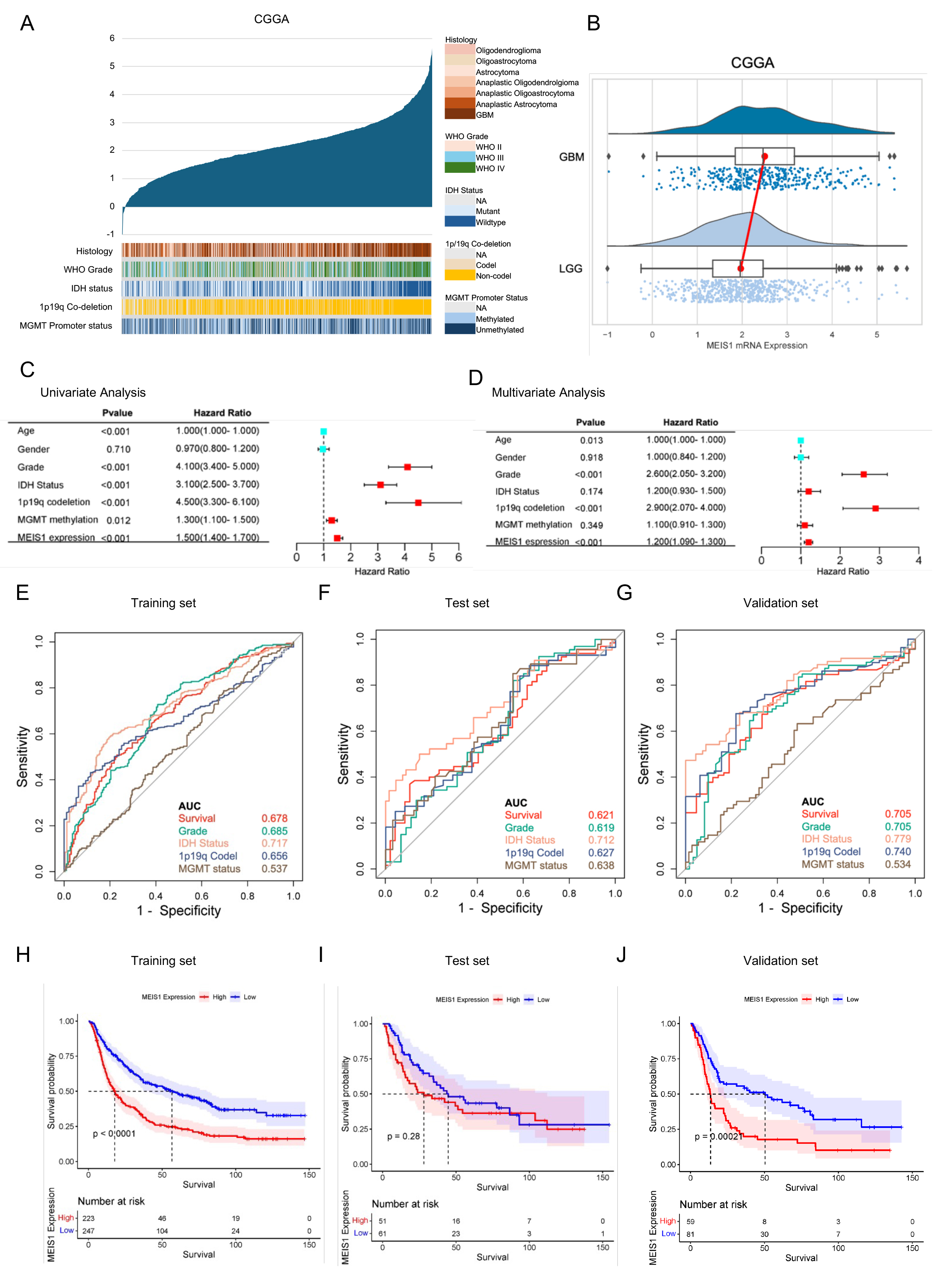

Supplement: Supplemental Material [file TACS_A_2327340_SM4634.zip › FigureS2.jpg]

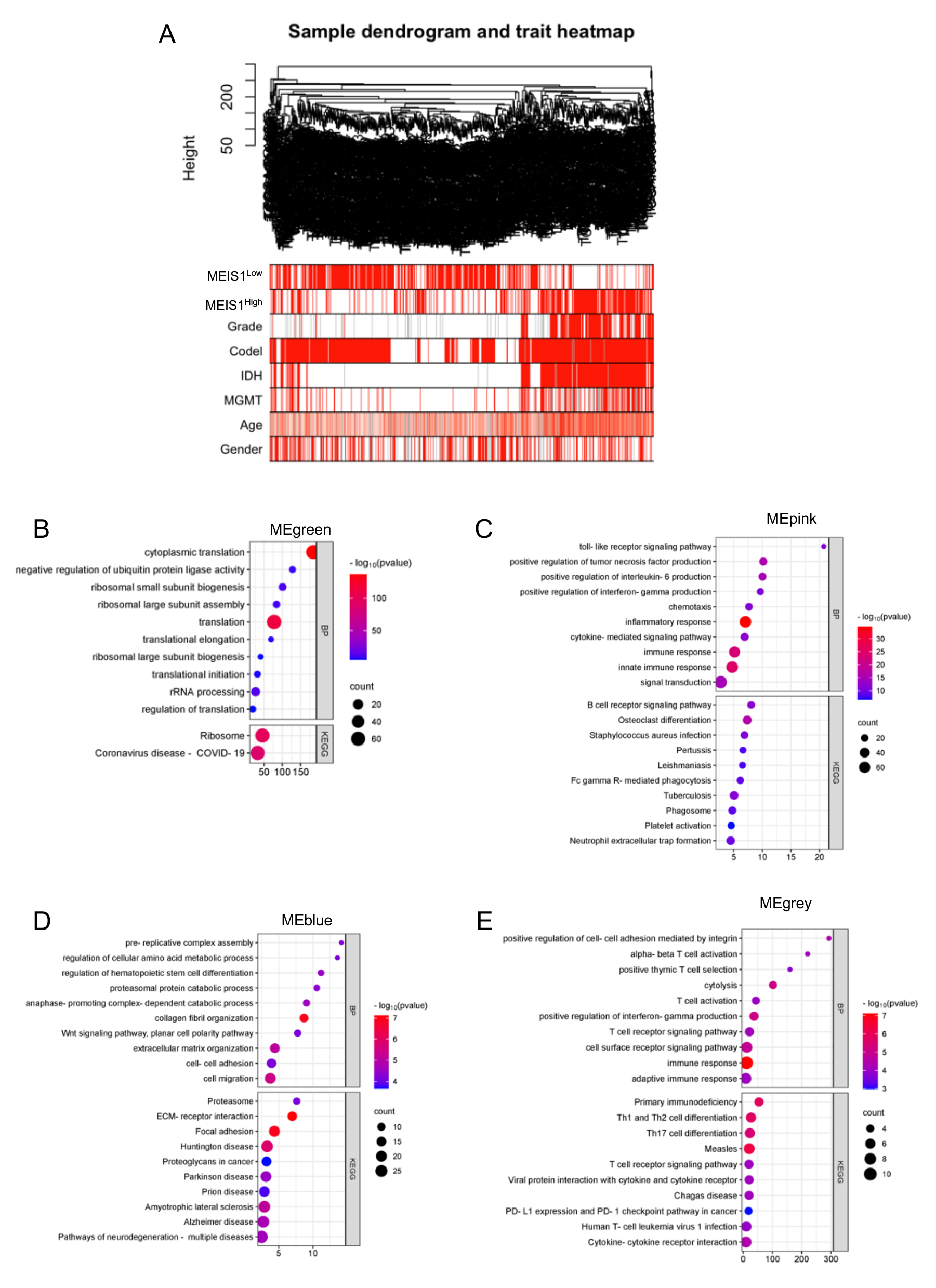

Supplement: Supplemental Material [file TACS_A_2327340_SM4634.zip › FigureS3.jpg]

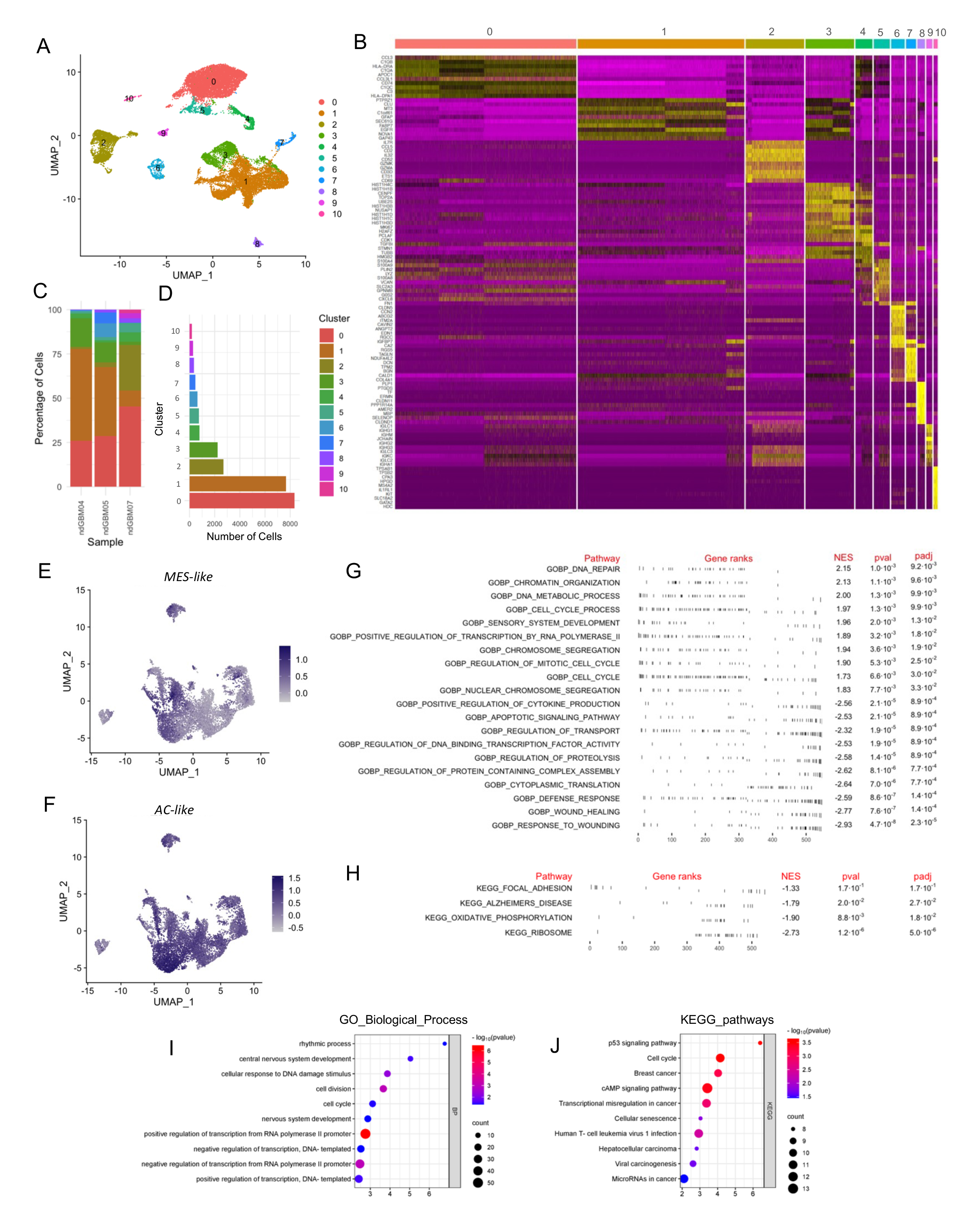

Supplement: Supplemental Material [file TACS_A_2327340_SM4634.zip › FigureS4.jpg]
